# Whole-genome characterization of prevalent dengue virus serotype-1 in 2023 dengue outbreak of Xishuangbanna, a border area of Laos, Myanmar, and China

**DOI:** 10.1016/j.ijregi.2025.100797

**Published:** 2025-11-04

**Authors:** Chenqian Peng, Tingting Li, Dehong Ma, Fen Zeng, Kaiyun Ding, Ziying Wu, Linhong Li, Yue Pan, Junying Chen, Yingcheng Guo, Qiangming Sun

**Affiliations:** 1 Institute of Medical Biology, Chinese Academy of Medical Sciences & Peking Union Medical College, Kunming, China; 2 National Kunming High-level Biosafety Primate Research Center, Institute of Medical Biology, Chinese Academy of Medical Sciences and Peking Union Medical College, Kunming, China; 3 Yunnan Provincial Key Laboratory of Vector-borne Diseases Control and Research, Kunming, China; 4 Xishuangbanna Dai Autonomous Prefecture People's Hospital, Xishuangbanna, China; 5 Xishuangbanna Dai Autonomous Prefecture Dai Hospital, Xishuangbanna, China; 6 Key Laboratory of Pathogen Infection Prevention and Control (Peking Union Medical College), Ministry of Education, Beijing, China; 7 State Key Laboratory of Respiratory Health and Multimorbidity, Beijing, China

**Keywords:** Dengue virus, Whole genome, Phylogenetic analysis, Mutation, Protein secondary structures

## Abstract

**Objectives:**

From June to November 2023, a severe dengue fever outbreak occurred in Xishuangbanna, China, a border area with Myanmar and Laos. This study aimed to identify the pathogen responsible for this outbreak and analyze the genomic characteristics of epidemic strains, providing reference data for dengue prevention and control.

**Methods:**

Serum samples from dengue virus (DENV) NS1-positive patients (July-October 2023) were collected. RNA extraction, serotyping via probe quantitative polymerase chain reaction, and whole-genome amplification using 18 primer pairs followed by sequencing were performed. Base and amino acid mutations were analyzed using DNAMAN. Phylogenetic trees (maximum likelihood for the whole genome and neighbor-joining for the E protein) were constructed using MEGA11. Protein secondary structures were compared via online tools provided by PRABI Lyon-Gerland.

**Results:**

Among 1465 samples, 833 were DENV-1 positive, with no other dengue serotype or flavivirus co-infection. Genomic analysis of 10 isolates showed high similarity to the 2023 Guangdong strain (PP540291). Non-structural proteins had higher base mutation rates than structural ones, with NS2 showing the highest (10.92%). Many unique 2013 Yunnan strain mutations were preserved. Phylogenetic trees clustered the epidemic strains with Guangdong and Southeast Asian isolates. NS1 RNA-binding sites remained stable.

**Conclusions:**

This study provides valuable insights for dengue control in the China-Myanmar-Laos border areas, as well as for viral pathogenesis research and vaccine development.

## Highlights

• A large-scale dengue fever outbreak occurred in the China-Laos-Myanmar border region in 2023.
• Dengue virus serotype 1 was the sole pathogen causing the 2023 dengue outbreak.
• 2023 Xishuangbanna dengue virus serotype 1 strains showed high homology to 2023 Guangdong isolates.
• Non-structural proteins (notably NS2) had higher mutation rates than structural ones.

## Introduction

Dengue virus (DENV) belongs to the *Flaviviridae* family and the *Flavivirus* genus and is classified into four serotypes (DENV-1 to DENV-4) based on the different antibodies produced following infection in humans [[1], [2], [3]]. DENV is a single-stranded, positive-sense RNA virus approximately 11 kb in length, consisting of an open reading frame (ORF) and two untranslated regions (5′ UTR and 3′ UTR) at both ends [4]. The translation of the ORF encodes three structural proteins (capsid [C], membrane [PrM/M], and envelope [E]) and seven non-structural proteins (NS1, NS2A, NS2B, NS3, NS4A, NS4B, and NS5) [5]. Dengue fever is a vector-borne infectious disease primarily transmitted by *Aedes aegypti*, prevalent in over 100 countries, particularly in urban and semi-urban areas of tropical and subtropical regions [[6], [7], [8]]. Research estimates that there are approximately 390 million dengue fever infections worldwide each year, representing a 30-fold increase over the past 50 years [9]. The first dengue fever outbreak in China was reported in Guangdong Province in 1978 [10], followed by subsequent outbreaks in other regions, including Hainan, Shanghai, Guangxi, Zhejiang, Fujian, and Taiwan. In 2008, dengue fever emerged in Ruili, a border city between China and Myanmar. By 2018, Southeast Asian countries bordering China reported dengue fever cases, including Vietnam, Laos, Cambodia, Myanmar, Thailand, and Malaysia. Notably, large-scale outbreaks occurred in Xishuangbanna, a tourist city in Yunnan Province adjacent to Laos and Myanmar, in 2013 and 2015 [11,12]. The local climate is highly conducive to the growth and reproduction of *Aedes aegypti*, making it one of the primary endemic areas for dengue fever. From 2013 to 2019, Xishuangbanna experienced small-scale regional outbreaks or new cases almost annually. The region's proximity to borders, unique climate, diverse vector species, and complex cross-border population dynamics pose significant challenges to DENV prevention and monitoring in China and neighboring countries [[13], [14], [15]]. In 2023, Yunnan Province witnessed its largest dengue fever outbreak on record, with a total of 13,483 cases reported, including 1180 imported cases (1069 from Myanmar, 100 from Laos, and 11 from five other countries) [11]. As a prefecture within Yunnan Province, Xishuangbanna shares geographical borders with Myanmar and Laos, reporting 7136 dengue cases in 2023 [16], resulting in an incidence rate of 545.64 per 100,000 population. This study aims to identify the pathogen responsible for the dengue fever outbreak and analyze the genomic characteristics of the epidemic strains during the 2023 outbreak in Xishuangbanna, providing reference data for dengue fever prevention and monitoring in this region and Southeast Asian countries.

## Materials and methods

### Collection of samples

During the DENV epidemic in the Xishuangbanna region in 2023, we collected serum samples from patients who tested positive for DENV-NS1 and sought medical treatment at the Xishuangbanna Dai Autonomous Prefecture People's Hospital from July to October 2023. A total of 2,250 serum samples (DENV-NS1 positive) were collected.

### DENV RNA extraction and serotyping

Total serum RNA was extracted using the QIAGEN QIAamp assay kit. Genotyping was performed using the probe quantitative polymerase chain reaction (qPCR) method with the Thermo Taqman kit. The probe primer sequences were designed according to the diagnostic guidelines for dengue fever established by the National Health Commission of the People's Republic of China. Four sets of probes and primers targeting DENV-1–4 were designed, along with four sets of identification primers for DENV, JEV, ZIKV, and CHIKV (Supplemental Table 1).

### Whole‐genome amplification and sequencing

Reverse transcription was conducted using the Takara PrimeScript RT reagent kit, resulting in the design of 18 pairs of primers for the polymerase chain reaction amplification of cDNA. The amplified products were subsequently sent to Bioengineering Biotech for sequencing, and the sequencing results were assembled to obtain the complete genome sequence.

### Analysis of molecular characteristics

Multiple sample sequences were compared with reference sequences using DNAMAN and MEGA11 software. A comprehensive analysis of base substitutions and amino acid mutation sites was performed, focusing on the molecular characteristics of the prM, E, and NS1 proteins. The primary sequences selected for analysis were those of type 1 DENV isolated in Yunnan Province, specifically KY672944 from 2013, MF405201 from 2015, MF681692 from 2017, MN639764 from 2018, and MW386902 from 2019. Additionally, the standard strain DQ672901 and the type 1 DENV sequence PP540291, isolated in Guangdong in 2023, were also included.

### Phylogenetic analysis

MEGA11 software was used to construct a maximum likelihood evolutionary tree of the whole genome and a neighbor-joining evolutionary tree of the E protein and to analyze the genetic relationship between the sequences in the evolutionary tree. The reference viral sequences were selected from the GenBank sequence database: AB178040 (Japan 2006), AB608788 (CNTaiWan 1994), AF226685 (Brazil 2007), AY722802 (Myanmar 1996), AY726555 (Myanmar 1998), AY762084 (Singapore 2004), DQ193572 (CNFuJian 2005), DQ672562 (Standard), EU081276 (Singapore 2005), EU848545 (Hawaii 1944), FJ176780 (CNGuangDong 2006), GQ357692 (Singapore 2008), HM469966 (Thailand 2007), HQ624984 (Cambodia 2007), JF459993 (Myanmar 2002), JN544411 (Singapore 2011), JQ045667 (Vietnam 2012), KC172835 (Laos 2008), KC672835 (Laos 2008), KF289072 (India 2011), KT827374 (CNGuangDong 2014), KU094071 (CNShenZhen 2015), KX380806 (Singapore 2013), KY672937 (CNYunNan 2014), KY849748 (Laos 2009), KY937185 (CNYunNan 2015), LC428080 (Vietnam 2017), LC652827 (Thailand 2015), MF033261 (Singapore 2016), MG679800 (CNYunNan 2017), MG679801 (CNYunNan 2017), MN448684 (Thailand 2012), MN912246 (Vietnam 2017), MT397277 (Thailand 2015), MW362477 (Thailand 2005), MW793710 (Myanmar 2019), MW386862 (CNYunNan 2019), MW386863 (CNYunNan 2019), MZ619041 (Thailand 2021), MZ857227 (Thailand 1992), OK469341 (Singapore 1992), OK469342 (Malaysia 1997), OK469343 (Pakistan 1994), OK469344 (Fiji 1975), OM281572 (CNShangHai 2018), ON038497 (Thailand 2013), OP121156 (Myanmar 2019), OQ426755 (Vietnam 2018), OQ832609 (Vietnam 2019), OQ832615 (Vietnam 2020), OR389016 (Vietnam 2008), PQ097697 (Thailand 2018), PP204095 (Vietnam 2023), PP268602 (Vietnam 2013), PP269457 (Vietnam 2011), PP269817 (Vietnam 2018), PP540291 (CNGuangDong 2023), PQ357532 (Vietnam 2017).

### Comparison of protein structures

In the context of protein-coding structure research, the prevalent strains identified in Xishuangbanna (CNYunNan 2023-1, CNYunNan 2023) and Guangdong Province (PP564728, CNGuangDong 2023) in 2023 were analyzed and compared to the standard DENV-1 strain DQ672562. The prediction of the protein secondary structure was conducted using online tools, with a specific focus on the nucleic acid-binding sites of the NS1 and NS5 proteins.

## Results

### Serotyping

In this study, a total of 1465 samples were tested for flavivirus co-infection and DENV serotype typing using probe qPCR method. No other flavivirus infections were detected, with 833 samples identified as type 1 positive and 632 samples identified as DENV negative.

### Whole-genome mutation analysis

Eighteen pairs of DENV-1 whole-genome amplification primers were utilized to amplify fragments from 10 viral strains, and full-length sequences were obtained through assembly using PCR and Sanger sequencing methods. The Xishuangbanna region, located in Yunnan Province, served as the study area. We selected representative sequences of DENV-1 strains (KY672944-2013 CNYunNan, MF405201-2017 CNYunNan, MF681692-2017 CNYunNan, MN639764-2019 CNYunNan, and MW386902-2019 CNYunNan) that have circulated in Yunnan in recent years for comparative analysis. Multiple sequence alignment was conducted using the built-in functions of SnapGene software under the MUSCLE strategy, focusing on these five sequences as well as two sequences from 2023. The results indicated that the base mutation rates were 7.31% (C), 8.03% (prM), 9.01% (E), 7.95% (NS1), 10.92% (NS2A, NS2B), 8.45% (NS3), 9.36% (NS4A, NS4B), and 8.20% (NS5). During the nucleic acid sequence alignment process, we observed that the non-structural proteins NS2 and NS4 exhibited higher mutation rate compared to other viral proteins. Furthermore, from a temporal perspective, KY672944 (2013 CNYunNan) harbored a significant number of unique mutation sites, suggesting that many subsequent mutations have been preserved and conferred an advantage in natural selection (Table 1).

**Table 1:** Analysis of the number of base mutation sites.

Structure protein	C	prM	E
CNYunNan2023-1	4	4	5
CNYunNan2023-2	4	4	5
PP540291 (2023-GuangDong, China)	4	4	5
KY672944 (2013-YunNan, China)	15	37	35
MF405201 (2017-YunNan, China)	3	4	4
MF681692 (2017-YunNan, China)	4	3	3
MN639764 (2019-YunNan, China)	4	3	3
MW386902 (2019-YunNan, China)	5	7	6


Non-Structure Protein	NS1	NS2A	NS2B	NS3	NS44A	NS4B	NS5
CNYunNan2023-1	6	5	5	6	3	3	8
CNYunNan2023-2	6	5	5	6	3	3	8
PP540291 (2023-GuangDong, China)	6	5	5	6	3	3	8
KY672944 (2013-YunNan, China)	40	34	29	38	25	28	40
MF405201 (2017-YunNan, China)	5	4	4	3	4	3	5
MF681692 (2017-YunNan, China)	4	3	3	2	3	3	5
MN639764 (2019-YunNan, China)	4	3	3	2	3	3	5
MW386902 (2019-YunNan, China)	5	6	5	5	4	4	7

**Figure 1:**
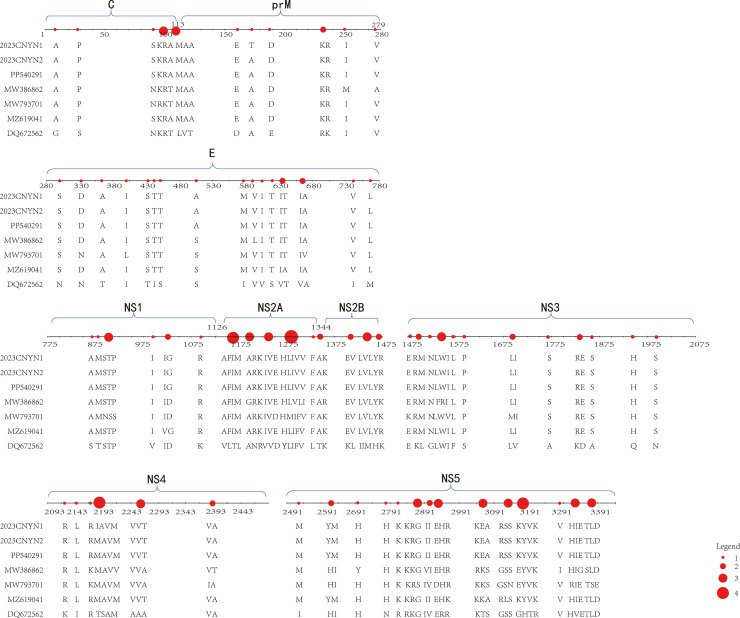
Amino acid sequence alignment results. CNYunNan2023-1 and CNYunNan2023-2 are sequences obtained in this study. PP540291 is the sequence from Guangdong Province, China, in the same year. DQ672562 was standard strain selected from the NCBI database. MW386862 is serotype 1 dengue virus sequence from Yunnan Province, 2019. MW793701 is serotype 1 dengue virus sequence from Myanmar, 2018. MZ619041 is serotype 1 dengue virus sequence from Thailand, 2021. They are all similar sequences to CNYNYunNan2023 according to the whole genome phylogenetic tree. The diameter of the dots is directly proportional to the number of mutation sites here.

The sequences in the table were selected from type 1 DENV isolates in Yunnan Province: KY672944 (2013), MF405201 (2015), MF681692 (2017), MN639764 (2018), and MW386902 (2019). Additionally, the type 1 DENV sequence PP540291, isolated in Guangdong in 2023, was also included. The numbers indicate unique mutations in the coding sequences of corresponding proteins, i.e., sites differing from the other six sequences. For CNYunNan 2023-1 and CNYunNan 2023-2, these refer to distinct sites compared to the other five sequences. Given the minimal sequence differences among the 10 prevalent DENV-1 strains in this region in 2023, we selected whole-genome sequences of two representative strains (CNYunNan 2023-1, CNYunNan 2023-2) and the standard strain (DQ672562) for comparison, focusing on base and amino acid mutations. Results (Figure 1) showed mutations across all encoded proteins, with amino acid mutation rates significantly lower than those of bases. This suggests that the virus accumulates numerous nonsense mutations during evolution, a hallmark of RNA viruses.

Statistics on the NS2A mutation rate of Yunnan DENV-1 strains from 2013 to 2023 showed: 10.2% for KY672944 (2013), 8.1% for MF405201 (2015), 8.3% for MF681692 (2017), 9.5% for MW386902 (2019), and 10.92% for 2023 strains, showing a slow upward trend. Notably, the 2023 strains had two novel NS2A mutations (T58A and R123K) not observed in post-2019 strains.

Subsequently, we selected 10 representative full-length strains with closely related geographical origins from recent years for base sequence and protein sequence alignment, focusing on the mutations and mutation frequencies of the structural proteins prM and E, as well as the non-structural proteins NS1 and NS5. In the protein sequences, prM exhibited nine mutations, E had 24 mutations, and NS1 presented 18 mutations. Table 2 provides a detailed list of mutation sites present in the prM protein, E protein, and NS1 protein, including mutations in the latest strain compared to the original strain and also mutations and reversion mutations in historical strains compared to the original strain.

**Table 2:** Variations among animo acid of DENV-1, especially show prM, E and NS1 proteins.

mutation sites	prM						E																				
	**6**						**21**																				
	169	173	232	236	250	275	288	317	332	394	429	435	441	451	462	505	577	592	604	617	619	649	662	666	719	741	764
DQ672562 (2001-Hawwi, Standard)	R	T	R	K	I	V	N	D	N	I	H	T	I	S	T	S	I	V	V	F	S	A	A	A	I	I	M
KU365900 (2014-TaiWan, China)	R	A	K	R	I	V	S	D	N	I	H	S	T	T	T	S	M	V	I	F	T	A	A	A	A	V	L
KT827374 (2014-GuangDong, China)	R	A	R	K	I	V	N	N	N	L	H	T	I	S	T	S	V	V	V	I	T	T	A	A	V	I	M
MF683116 (2017-YunNan, China)	R	A	K	R	I	V	S	D	N	I	H	S	T	T	K	S	M	V	I	F	T	A	A	A	I	V	L
MG679800 (2017-YunNan, China)	R	A	K	R	I	V	S	D	N	L	H	S	T	T	T	S	M	V	I	F	T	A	V	A	I	V	L
MG679801 (2017-YunNan, China)	R	A	K	R	I	V	S	D	N	L	H	S	T	T	T	S	M	V	I	F	T	A	V	A	I	V	L
MW386862 (2019-YunNan, China)	Q	T	K	R	M	A	S	D	D	I	H	S	T	T	T	S	M	L	I	F	T	A	A	A	I	V	L
MW386863 (2019-YunNan, China)	Q	T	K	R	M	V	S	D	D	I	Y	S	T	T	T	S	M	L	I	F	T	A	A	T	I	V	L
PP540291 (2023-GuangDong, China)	R	A	K	R	I	V	S	D	D	I	H	S	T	T	T	A	M	V	I	F	T	A	A	A	I	V	L
CNYunNan2023-1	R	A	K	R	I	V	S	D	D	I	H	S	T	T	T	A	M	V	I	F	T	A	A	A	I	V	L


mutation sites	NS1														
	**15**														
	869	873	874	892	903	906	914	919	937	953	995	###	###	###	###
DQ672562 (2001-Hawwi, Standard)	S	A	Q	S	V	T	N	P	I	V	V	K	D	V	K
KU365900 (2014-TaiWan, China)	A	A	Q	S	V	T	N	P	I	V	V	K	D	V	K
KT827374 (2014-GuangDong, China)	N	T	Q	S	T	T	D	P	V	M	V	R	N	V	R
MF683116 (2017-YunNan, China)	A	A	Q	S	V	T	N	P	I	V	V	K	D	F	K
MG679800 (2017-YunNan, China)	A	A	R	N	V	S	N	S	I	V	D	K	D	V	K
MG679801 (2017-YunNan, China)	A	A	Q	N	V	S	N	S	I	V	V	K	D	V	K
MW386862 (2019-YunNan, China)	A	A	Q	S	V	T	N	P	I	V	V	K	D	V	K
MW386863 (2019-YunNan, China)	A	A	Q	S	V	T	N	P	I	V	V	K	D	V	K
PP540291 (2023-GuangDong, China)	A	A	Q	S	V	T	N	P	I	V	V	K	G	V	K
CNYunNan2023-1	A	A	Q	S	V	T	N	P	I	V	V	K	G	V	K

The letters are abbreviations of single characters corresponding to amino acids. KU365900 is from Taiwan Province of China from 2014, and KT 827374 is from Guangdong Province of China from 2014. They present the DENV-1 of China 10 years ago. MF683116, MG679800, MG679801 are from Yunnan Province of China from 2017, and MW386862, MW386863 are from Yunnan Province of China from 2019. They present the DENV-1 in Yunnan Province from recent years. PP540291 is from Guangdong Province of China from 2023, showing the different outbreak of dengue virus serotype 1 in China in 2023.

DENV-1, dengue virus serotype 1.

A total of 10 DENV-1 strains were isolated from patient sera in Xishuangbanna in 2023. In addition to CNYunNan 2023-1 and CNYunNan 2023-2 (focused on in this study), full-genome sequencing of the remaining eight strains (named CNYunNan 2023-3 to CNYunNan 2023-10) has been completed via Sanger sequencing, and the sequences are currently being submitted to GenBank (provisional accession numbers: PV627872–PV627881). Sequence alignment showed 99.2%–99.8% nucleotide homology and 99.5%–99.9% amino acid homology among the 10 strains, with no significant genetic differences (≤20 variant sites). This confirms that the 2023 Xishuangbanna DENV-1 outbreak was caused by a single viral cluster and that CNYunNan 2023-1 and CNYunNan 2023-2 are representative of the circulating strains from that year.

### Phylogenetic analysis

**Figure 2:**
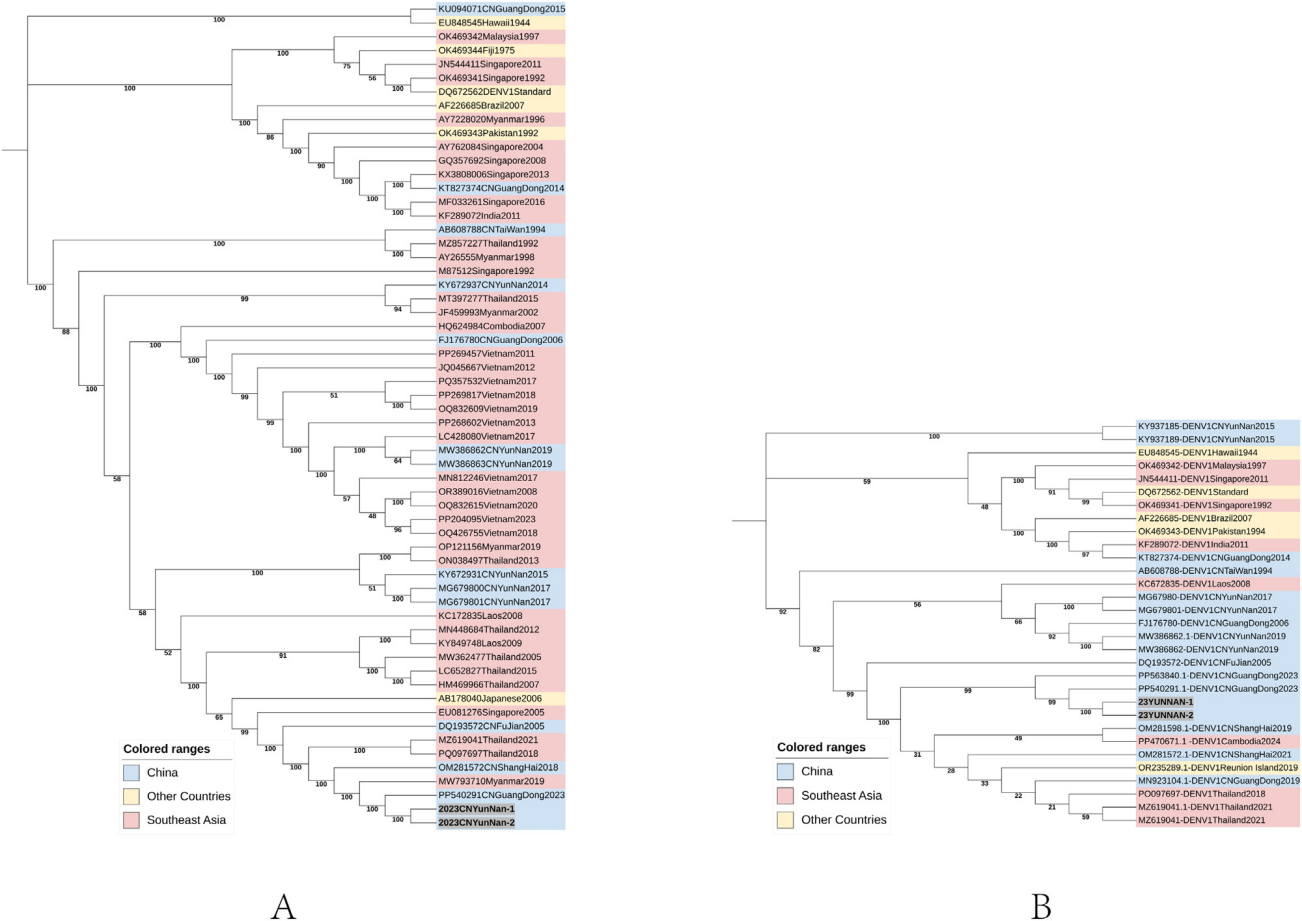
Phylogenetic tree of DENV-1 epidemic strains in Xishuangbanna, Yunnan, China, in 2023. The dark marked strains are the ones sequenced this research.

The viral genome evolutionary tree constructed using the maximum likelihood method (Figure 2) indicates that the DENV type 1 strain PP540291, isolated in Guangdong, China in 2023, belongs to the same branch cluster as the strain obtained in this study. Furthermore, the strain that is relatively close in China is OM281572, a DENV type 1 strain isolated in Shanghai, China in 2018. Given the proximity of Xishuangbanna to Southeast Asian countries, the DENV type 1 strains prevalent in these countries in recent years also exhibit similarities to the strains obtained in this study, including the 2019 Myanmar epidemic strain MW793710, as well as the 2017 and 2021 epidemic strains PQ097697 and MZ619041. The two strains of the virus isolated in this study formed distinct clusters, with both viruses from Guangdong in the same year positioned in the two most adjacent clusters. These four viruses form a cluster adjacent to another cluster predominantly composed of Southeast Asian strains. The results suggest that the source of the dengue fever outbreak primarily originates from the prevalent strains in Southeast Asia. By selecting the closely related strain PP540291 for base sequence and amino acid sequence alignment, it was observed that there are fewer than 20 amino acid differences.

### Comparison of viral protein structures

**Figure 3:**
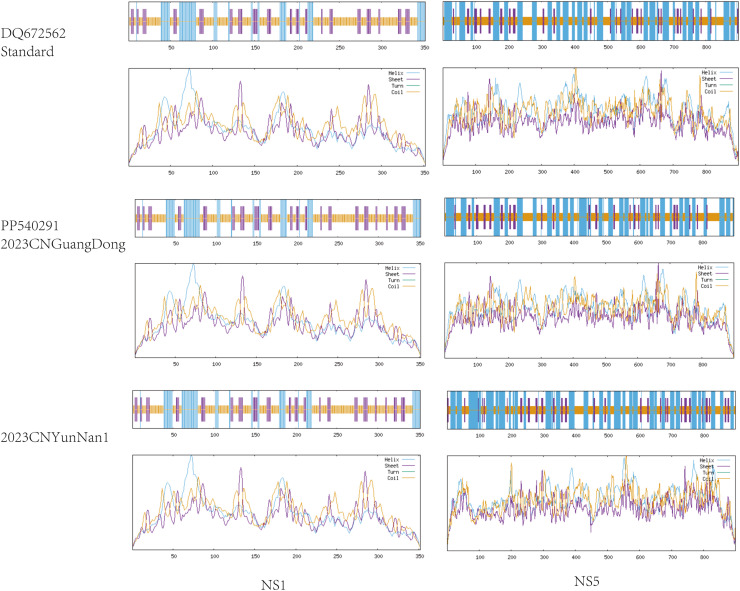
Predicted secondary structures of non-structural proteins (NS1 and NS5) of DENV-1” to avoid ambiguity.

This study focuses on analyzing the coding regions of CNYunNan 2023-1, CNYunNan2023-2, PP564728 (CNGuangDong 2023), and the DENV-1 standard strain DQ672562. We employed the SOPMA secondary structure prediction tool provided by PRABI Lyon-Gerland to compare protein secondary structures, with special attention to the nucleic acid-binding sites of NS1 and NS5 proteins. Since NS1 is involved in viral replication, our analysis of its secondary structure specifically focused on variations in nucleic acid-binding sites. Compared with the 2023 strains, the RNA-binding sites of NS1 remain stable at four (Figure 3). The relative stability of these RNA-binding sites may be attributed to the inherent nature of the virus as an RNA virus.

Figure 3 showed the prediction of the secondary structures of non-structural proteins (NS1 and NS5) of DENV-1. Secondary structure prediction was conducted using the SOPMA (Self-Optimized Prediction Method with Alignment) tool from the PRABI Lyon-Gerland online platform, with parameters set as follows: window size = 17, similarity threshold = 8, and predicted structure types including α-helix, β-sheet, random coil, and β-turn. Analysis focused on NS1 and NS5. The RNA-binding site (amino acids 103–120) of NS1 exhibited an α-helix structure, which was identical between the 2023 strains and the standard strain DQ672562, indicating that mutations did not affect its RNA-binding function. The I325L mutation in NS5 was located in a random coil region without key functional domains (e.g., RNA polymerase active center), suggesting no impact on NS5’s RNA replication activity. NS1 showed more conserved secondary structure than NS5, likely due to its core role in viral immune evasion.

## Discussion

DENV-1 has long exhibited a relatively widespread prevalence in Southeast Asia. Over the past decade, DENV-1, DENV-2, and DENV-3 have remained the predominant serotypes in dengue fever outbreaks, whereas DENV-4 has been less frequently observed in the region [17]. Phylogenetic analysis revealed that the DENV-1 strain isolated from patient sera during the 2023 epidemic in Xishuangbanna exhibited the closest genetic relatedness to the strain isolated from Guangzhou cases in the same year, followed by strains prevalent in Thailand in 2017 and 2021, as well as a strain isolated in Shanghai in 2018 that is also closely related. Considering the phylogenetic tree of the E protein-coding sequence, which exhibits relatively conservative evolutionary characteristics, all epidemic strains in the closest cluster in this study are strains that have been prevalent in China and Southeast Asian countries over the past 5 years. This not only indicates that it can remain relatively stable as an envelope protein in evolution, but also confirms that the selected mutations can be preserved relatively stable and may gradually become predominant in the population [18,19].

Whole-genome phylogenetic tree analysis showed that DENV strains circulating in Vietnam clustered more tightly, whereas those from China were dispersed across different clades. This suggests that viruses emerging in Vietnam exhibit closer evolutionary relatedness, with relatively stable and conserved genetic characteristics. In contrast, strains spreading in China display more diverse evolutionary origins and carry a higher frequency of mutations. Focusing on the amino acid mutations of the prM protein, E protein, and NS1 protein (Table 2), the two epidemic strains, 23YN and 23GD, exhibit high sequence similarity, indicating they likely share the same origin. Given the earlier dengue outbreak in Xishuangbanna, we hypothesize that the Guangzhou epidemic virus was introduced by tourists returning from Xishuangbanna. Notably, an intriguing phenomenon was observed: amino acid mutations at certain loci initially shifted from the ancestral residues to other amino acids, but then reverted to the ancestral state in the most recent strain, providing clear evidence of revertant mutations.

PrM is the membrane protein of the DENV, and the E protein is the envelope protein. Although NS1 and NS5 are non-structural proteins, their functions are closely related to viral RNA replication and pathogenesis. This indicates that these proteins are closely associated with the infectivity, replication, and pathogenicity of the virus. Proteins crucial for the survival of viruses tend to have relatively conserved amino acid sequences. This is particularly true for RNA viruses, which often exhibit numerous mutations in their genomes, the vast majority of which are nonsense mutations, with only a few genuinely affecting the amino acid sequence. Furthermore, among the mutated amino acids, mutations such as T→A, R→ K, and I→ L do not cause significant changes in protein structure due to the similarity in polarity between the two amino acids. Comparative analysis of the sequences revealed that most mutations are similar, with a few occurrences of mutations that could severely impact protein structure. It can be inferred that these mutations have been eliminated through natural selection and cannot be retained during viral replication. NS1 is an important non-structural protein that not only participates in viral replication but also in viral immune evasion and other related processes. Analysis of NS1 indicates the presence of missense mutations in its coding sequence; however, its secondary structure remains relatively stable, particularly at the RNA-binding site, which has shown consistent stability even after decades of variation, aligning with the original strain. NS5 is a crucial non-structural protein believed to primarily function as an RNA-dependent RNA polymerase and also participate in methylation processes [20]. This study found that, compared to NS1, NS3, and NS4, NS5 appears to have more mutation sites in both the base sequence and amino acid sequence, exhibiting a higher mutation rate. These mutations facilitate the virus strain's adaptation to the continually evolving host environment, allowing it to become the dominant strain through natural selection and leading to epidemic outbreaks. Therefore, NS5 can be considered a target for developing specific drugs for dengue fever, aimed at inhibiting the virus's replication and proliferation [21,22].

NS2A is a key component of the DENV replication complex, participating in viral RNA replication and host immune evasion [23]. The high NS2A mutation rate (10.92%) and novel mutations (T58A, R123K) in the 2023 strains may enhance interactions with host cytokines: The T58A mutation may improve NS2A’s binding efficiency to the host endoplasmic reticulum membrane, promoting the assembly of the viral replication complex. The R123K mutation (a polar-conservative amino acid substitution) may maintain protein structural stability while reducing recognition of the virus by host innate immune molecules (e.g., interferon-β). Such adaptive mutations may have enhanced the virus’s transmissibility in the population, providing a molecular basis for the large-scale 2023 Xishuangbanna outbreak. Additionally, the increasing NS2A mutation rate highlights the need for continuous monitoring of its evolutionary trend to inform dynamic targets for vaccine design.

Among the mutations observed in the 23YN strain, the substitution from serine to alanine at position 296 of the envelope (E) protein (if counted from C, it is located at S505A) stands out as particularly noteworthy for its potential impact on viral epidemiology. This S505A mutation was uniquely identified in the 23YN strain and a contemporaneous 23GZ strain, but not in the standard reference strain or earlier circulating strains from our dataset (such as 14TW, 17YN, and 19YN). The convergent emergence of this mutation in two recent and distinct lineages suggests that it may confer a selective advantage, potentially contributing to the successful circulation of these newer variants.

The S505A (E-S296A) mutation of the isolated epidemic strain resides within the soluble ectodomain (sE, residues 1-395) of the DENV E protein, near the kl hairpin (residues 270-279, a pH-sensitive hinge for Domain I-II rotation) and the Domain I-III interface—both critical for E protein conformational transitions and trimer assembly during membrane fusion [24].

Serine (S) at position 296 likely contributes to local structural stability via hydroxyl-mediated hydrogen bonding; alanine (A), lacking this polar group, may disrupt nearby folding. For infectivity, this could impair pH-triggered dimer dissociation or trimer formation: compromised kl hairpin flexibility or Domain I-III interactions might reduce fusion loop insertion into host membranes, affecting viral entry efficiency. For pathogenicity, reduced infectivity may decrease viral load, mitigating disease severity. However, if the mutation alters trimer stability without abolishing fusion, it might indirectly affect immune recognition via subtle E protein structural changes.

In summary, the source of the DENV-1 strain in Xishuangbanna in 2023 is relatively singular and shows strong similarities with strains that have long been present in Southeast Asia and southern China. Consequently, it is imperative to enhance prevention and control measures for mosquito vectors and to strengthen external surveillance of cross-border individuals to strictly prevent the importation of cross-border strains. In 2023, dengue fever outbreaks occurred in many parts of China and numerous countries worldwide. Conducting such virus genome analyses not only aids in tracing the origin of the virus and analyzing the causes and formation of virus epidemics but also provides insights and assistance for the prevention and treatment of dengue fever. Additionally, it offers a reference for the development of candidate vaccines, analyzes the impact of various site mutations on virus virulence, and screens attenuated candidate strains with potential for vaccine development. Despite the rapid global spread of DENV infection in recent years, leading to millions of infections annually, there remains a lack of approved DENV vaccines or specific antiviral drugs and treatments to control the DENV epidemic.

Due to the unique geographical conditions of Xishuangbanna, dengue fever outbreaks occur almost biennially in the region, particularly in 2019 before the COVID-19 pandemic and in 2023 following the lifting of COVID-19 population control measures. This study provides a reliable reference for understanding the mechanisms and treatment strategies for dengue infection, which is crucial for addressing the treatment challenges of dengue fever and developing effective DENV vaccines.

This study has the following limitations: viral isolation and sequencing were performed only on serum samples from patients, and no concurrent collection of local mosquito vectors, primarily *Aedes aegypti,* was carried out in Xishuangbanna, which precludes direct verification of whether homologous DENV-1 strains were present in mosquitoes or analysis of adaptive mutations of the virus during the human–mosquito transmission cycle. Furthermore, the lack of viral sequence data from cross-border mobile populations, such as imported cases from Myanmar and Laos, impedes accurate tracing of the cross-border transmission pathway of the outbreak strain. This highlights the need for future studies that integrate mosquito vector surveillance with cross-border case sequencing to reconstruct a complete human–mosquito–cross-border transmission chain and thereby provide more precise support for dengue prevention and control in border areas.

## Funding

This research was supported by Foundation of the CAMS Initiative for Innovative Medicine (2021-I2M-1-036), Major Basic Research Projects of Yunnan Science and Technology Department (202401BC070008),Major Science and Technology Projects of Yunnan Science and Technology Department (202402AA310021), Yunling Scholar Talent Project of Yunnan Province (YNWR-YLXZ-2019-008).

## Ethics statement

Ethical approval was obtained from the Institutional Ethics Committee (Xishuangbanna Dai Autonomous Prefecture People's Hospital).

## Author contributions

Chenqian Peng and Qiangming Sun designed the research. Fen Zeng, Ziying Wu,L inhong Li, Yingcheng Guo, Dehong Ma and Tingting Li collected blood samples and clinical detection. Kaiyun Ding, Yue Pan, Junying Chen provided the study materials. Chenqian Peng performed the experiment. Chenqian Peng wrote the manuscript and analyzed the data. Qiangming Sun performed the article revision, and editing the manuscript.

## Declaration of competing interest

The authors have no competing interests to declare.

## Appendix Supplementary materials

Image, application 1

Image, application 2

Image, application 3
