# Supplementary material for: Whole-genome characterization of prevalent dengue virus serotype-1 in 2023 dengue outbreak of Xishuangbanna, a border area of Laos, Myanmar, and China
Source: IJID Reg. 2025 Nov 4;17:100797. doi: 10.1016/j.ijregi.2025.100797 (PMC12704074; doi:10.1016/j.ijregi.2025.100797)
Supplement: Supplementary file 1 [file mmc1.pdf]

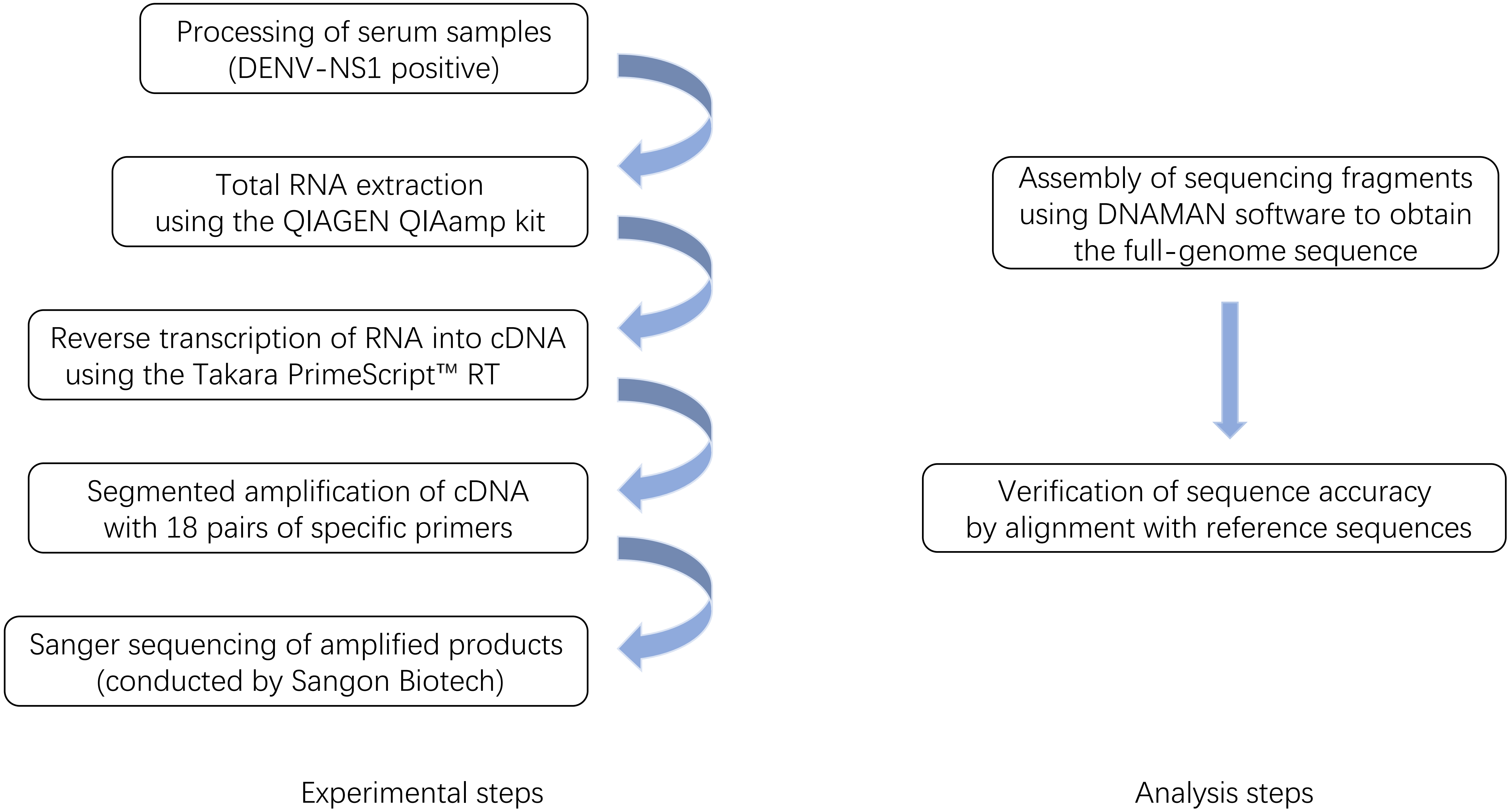
Processing of serum samples
(DENV-NS1 positive)
Total RNA extraction
using the QIAGEN QIAamp kit
Reverse transcription of RNA into cDNA
using the Takara PrimeScript™ RT
Segmented amplification of cDNA
with 18 pairs of specific primers
Sanger sequencing of amplified products
(conducted by Sangon Biotech)
Assembly of sequencing fragments
using DNAMAN software to obtain
the full-genome sequence
Verification of sequence accuracy
by alignment with reference sequences
Experimental steps
Analysis steps
